# Protective Effects of Licorice (*Glycyrrhiza uralensis*) Against Vancomycin-Induced Nephrotoxicity In Vivo and In Vitro

**DOI:** 10.3390/ph19050728

**Published:** 2026-05-04

**Authors:** Jianping Zhang, Yan Zhou, Ruirui Cui, Lijun Wang, Sijia Wang, Wenhan Rao, Xinan Wu

**Affiliations:** 1 Department of Pharmacy, The First Hospital of Lanzhou University, Lanzhou 730000, China; zhangjianping0306@163.com (J.Z.); zhouyan21@lzu.edu.cn (Y.Z.); ruicui98@163.com (R.C.); wanglijun391@163.com (L.W.); 2 Engineering Research Center of Prevention and Control for Clinical Medication Risk, Lanzhou 730000, China; 3 The First School of Clinical Medicine, Lanzhou University, Lanzhou 730000, China; 320220908760@lzu.edu.cn (S.W.); 320220911191@lzu.edu.cn (W.R.)

**Keywords:** licorice, protective effect, vancomycin, nephrotoxicity, oxidative stress, mitochondria, inflammation, intestinal microbiota, short chain fatty acids, uremic toxins

## Abstract

**Background:** Vancomycin (VAN)-induced nephrotoxicity limits its clinical application. Licorice (*Glycyrrhiza uralensis* Fisch.) and its bioactive constituents have been reported to protect against nephrotoxicity induced by various nephrotoxic agents. This study aimed to evaluate the protective effects of licorice against VAN-induced nephrotoxicity and to explore the underlying mechanisms both in vivo and in vitro. **Methods:** Seven groups of male C57BL/6 mice received different treatments for 7 consecutive days. Blood, fecal and renal tissue samples were collected for the assessment of serum creatinine, renal histopathology, mitochondrial ultrastructure, oxidative stress markers, kidney injury molecule-1 (Kim-1), short-chain fatty acids (SCFAs), and uremic toxins. In human proximal tubular epithelial cells (HK-2 cells), the effects of licorice on cell viability, oxidative stress, inflammatory markers, and mitochondrial membrane potential (MMP) were further investigated. **Results:** Licorice significantly attenuated VAN-induced nephrotoxicity and restored glutathione peroxidase (GSH-Px) activity while reducing malondialdehyde (MDA) levels. In addition, licorice markedly ameliorated VAN-induced renal histopathological injury, as demonstrated by hematoxylin and eosin staining and transmission electron microscopy. Licorice also reversed VAN-induced intestinal microbiota dysbiosis and increased the relative abundance of SCFA-producing bacteria, including *Bacteroides*. Moreover, licorice treatment increased fecal SCFA contents and modulated multiple uremic toxins in both serum and renal tissue. Consistently, licorice protected HK-2 cells against VAN-induced cytotoxicity by regulating GSH, interleukin-1β (IL-1β), interleukin-6 (IL-6), tumor necrosis factor-α (TNF-α), and MMP. **Conclusions:** These findings demonstrate that licorice exerts protective effects against VAN-induced nephrotoxicity in vivo and in vitro, suggesting the potential involvement of oxidative stress, mitochondrial structure and function, inflammation, intestinal microbiota-SCFAs and uremic toxins.

## 1. Introduction

Vancomycin (VAN) is a first line agent for the treatment of methicillin-resistant *Staphylococcus aureus* infections; however, its nephrotoxicity limits its clinical application. VAN-induced nephrotoxicity may present as acute interstitial nephritis or acute tubular necrosis [1]. The reported incidence of VAN-associated acute kidney injury (VAN-AKI) varies across studies. In a prospective, multicenter observational study including 265 patients with methicillin-resistant *Staphylococcus aureus* bacteremia treated with VAN, the incidence of AKI was 26% [2]. In contrast, a retrospective observational cohort study from China involving patients with infective endocarditis who received VAN reported an AKI incidence of 14.3% [3]. VAN-AKI is associated with prolonged hospitalization, increased hospital readmission, and higher mortality [4]. The pathophysiological mechanisms of VAN-AKI include oxidative stress [5], VAN-associated cast nephropathy [6]. As effective conventional treatments for VAN-AKI remain limited, prevention is still considered the most important strategy [7]. Pais, G.M. et al. [1] summarized several potential preventive approaches for VAN-induced nephrotoxicity, including antioxidant therapy, hydration, the administration of transport inhibitors to reduce cellular accumulation, and formulation modifications to alter renal clearance mechanisms. Among these strategies, antioxidants have been the most extensively investigated. A variety of antioxidants, including astaxanthin [8], rutin [9], naringenin [10], zingerone [11], chlorogenic acid [12], rhein [13], scopoletin [14], silymarin [15], *Silybum marianum* extract [16], pomegranate (*Punica granatum*) peel ethanolic extract [17], and hydroalcoholic extract of *Rosa canina* fruit [18], have been reported to attenuate oxidative stress and alleviate VAN-induced nephrotoxicity.

Licorice has been widely used medicinally in China since ancient times. It is frequently combined with other traditional Chinese medicines (TCMs) in classical prescriptions; notably, its occurrence rate in *Shanghan Lun* reached 61.9% [19]. Another study reported that licorice appeared 26,185 times in the Chinese Formula Database, which contains 96,592 formula records, suggesting its extensive use in TCMs for attenuating toxicities and enhancing pharmacological effects [20]. Accordingly, numerous licorice-containing formulations have been developed into patented medicines in China [21].

Licorice and its bioactive constituents are known to exert a broad range of pharmacological activities, including antioxidant, anti-inflammatory, and hepatoprotective effects. In addition, protective effects of licorice against nephrotoxicity induced by doxorubicin, cisplatin, lead, cadmium, acyclovir, and gentamicin have been reported [22]. For example, *Glycyrrhiza glabra* root extract ameliorated gentamicin-induced renal injury, likely through its antioxidant and anti-inflammatory properties [23]. Moreover, molecular docking analysis showed that liquiritigenin and liquiritin can bind to Kelch-like ECH-associated protein 1 (KEAP1), a key regulator of the transcriptional activity of nuclear factor erythroid 2-related factor 2 (Nrf2) [24].

Oral administration is the most common route of licorice use; therefore, the intestinal microbiota is among the first biological systems exposed to licorice and its constituents. Accumulating evidence has demonstrated that licorice and its active ingredients can significantly modulate the composition and function of the intestinal microbiota [25,26,27]. The intestinal microbiota is closely associated with the production of uremic toxins [28] and short-chain fatty acids (SCFAs) [29], and it also plays an important role in renal physiology and pathological progression. Based on these findings, we hypothesized that licorice may alleviate VAN-induced nephrotoxicity by regulating the intestinal microbiota. However, whether the intestinal microbiota is indeed involved in the renoprotective effects of licorice against VAN-induced nephrotoxicity remains unclear.

The novelty of the present study lies in its comprehensive and systematic investigation of the protective effects and underlying mechanisms of licorice against VAN-induced nephrotoxicity, both in vivo and in vitro. Multiple key aspects were evaluated, including oxidative stress, mitochondrial structure and function, renal histopathology, cell viability, and inflammation. Notably, we further investigated alterations in the intestinal microbiota, short-chain fatty acids (SCFAs), and uremic toxin accumulation. Collectively, this study provides a more comprehensive and novel insight into the renoprotective effects of licorice and the multitarget mechanisms by which it attenuates VAN-induced nephrotoxicity.

## 2. Results

### 2.1. Quality Control of Licorice Aqueous Extract

Licorice was extracted independently in triplicate, and high-performance liquid chromatography (HPLC) was used to determine the presence of glycyrrhizic acid, liquiritin, and isoliquiritigenin. As shown in Figure S1, the retention times of these three constituents were highly consistent with those of the corresponding reference standards, confirming their presence in the aqueous extract of licorice. The linear ranges of the standard curves for glycyrrhizic acid, liquiritin, and isoliquiritigenin were 4.14–132.5, 1.88–60, and 0.7–45 μg/mL, respectively. The linear correlation coefficients (*R*^2^) of all three compounds were greater than 0.999, indicating excellent linearity. The contents of glycyrrhizic acid, liquiritin, and isoliquiritigenin in raw licorice were 2.79 ± 0.36%, 0.66 ± 0.13%, and 0.11 ± 0.01%, respectively.

Liquid chromatography-tandem mass spectrometry (LC-MS/MS) was used to analyze liquiritin, isoliquiritigenin, quercetin, liquiritin apioside, glycyrrhizic acid, and glycyrrhetinic acid in the aqueous extract of licorice. Among these compounds, liquiritin, isoliquiritigenin, quercetin, and liquiritin apioside were quantitatively determined in triplicate, and the corresponding chromatograms and mass spectra are shown in Figure S2. The linear ranges of the standard curves for liquiritin, isoliquiritigenin, quercetin, and liquiritin apioside were 1.95–500, 1.95–1000, 1.95–1000, and 1.95–1000 ng/mL, respectively. The linear correlation coefficients (*R*^2^) for all four compounds were greater than 0.999, indicating excellent linearity. The contents of liquiritin, isoliquiritigenin, quercetin, and liquiritin apioside in raw licorice were 0.71 ± 0.07%, 0.13 ± 0.01%, 0.014 ± 0.001%, and 0.52 ± 0.06%, respectively.

### 2.2. Licorice Improves VAN-Induced Nephrotoxicity in Mice

**Figure 1:**
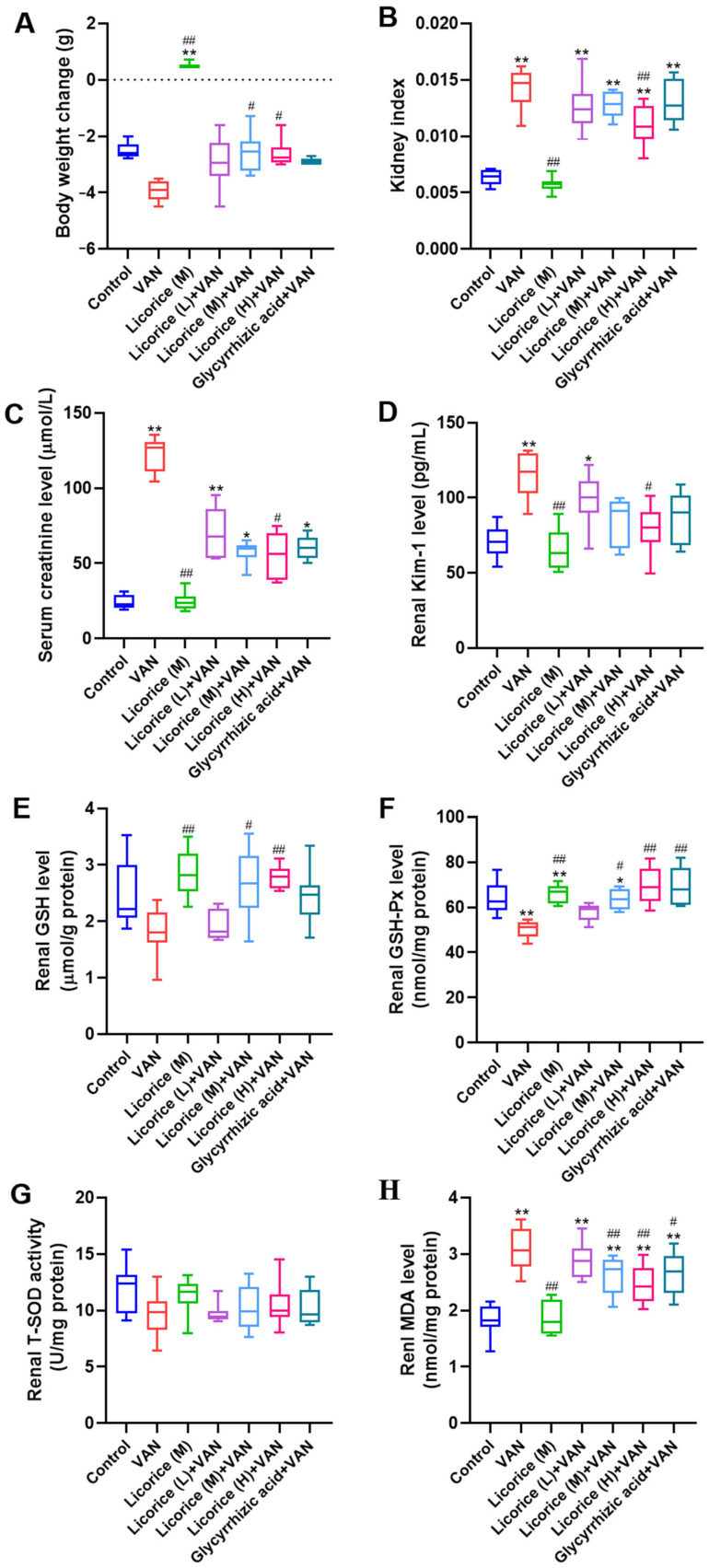
Effects of licorice on VAN-induced nephrotoxicity in mice (*n* = 10). (**A**) Body weight change; (**B**) kidney index; (**C**) serum creatinine level; (**D**) renal kidney injury molecule-1 (Kim-1) level; (**E**) renal glutathione (GSH) level; (**F**) renal glutathione peroxidase (GSH-Px) activity; (**G**) renal total superoxide dismutase (T-SOD) activity; and (**H**) renal malondialdehyde (MDA) level. The doses of Licorice (L), Licorice (M), and Licorice (H) were 0.4, 2, and 10 g raw licorice/kg body weight, respectively, administered twice daily to mice. Data are presented as box-and-whisker plots (median, interquartile range, minimum-maximum). Nonparametric testing (Kruskal–Wallis H test followed by Dunn’s post hoc test) was used to analyze body weight change, serum creatinine, renal Kim-1, GSH, and GSH-Px levels. Kidney index data were analyzed by one-way ANOVA followed by Tamhane’s T2 post hoc test. MDA levels were analyzed by one-way ANOVA followed by Dunnett’s post hoc test. ** *p* < 0.01, * *p* < 0.05 vs. the control group; ## *p* < 0.01, # *p* < 0.05 vs. the VAN group.

Compared with the control group, body weight was markedly reduced in the VAN group (Figure 1A), whereas VAN administration significantly increased the kidney index (Figure 1B). Compared with the VAN group, treatment with all doses of licorice and glycyrrhizic acid tended to attenuate VAN-induced body weight loss and the increase in kidney index. Notably, only the Licorice (M) + VAN and Licorice (H) + VAN groups significantly alleviated body weight loss (*p* < 0.05), while only the Licorice (H) + VAN group significantly reduced the kidney index (*p* < 0.01) relative to the VAN group.

Serum creatinine levels (Figure 1C) and renal kidney injury molecule-1 (Kim-1) expression (Figure 1D) were significantly elevated in the VAN group, indicating severe VAN-induced nephrotoxicity. Treatment with all doses of licorice and glycyrrhizic acid reduced serum creatinine levels compared with the VAN group, with a statistically significant reduction observed in the Licorice (H) + VAN group (*p* < 0.05). Consistent with the creatinine findings, all intervention groups showed a trend toward attenuating the increase in Kim-1, and a significant reduction was observed in the Licorice (H) + VAN group (*p* < 0.05) compared with the VAN group.

As illustrated in Figure 1E–H, VAN exposure induced pronounced oxidative stress in renal tissue, as evidenced by a marked reduction in antioxidant biomarkers and increased lipid peroxidation products compared with the control group. Specifically, the VAN group exhibited a significant decrease in renal glutathione peroxidase (GSH-Px) activity (Figure 1F, *p* < 0.01), accompanied by downward trends in glutathione (GSH) content (Figure 1E) and total superoxide dismutase (T-SOD) activity (Figure 1G), although these changes did not reach statistical significance. In parallel, renal malondialdehyde (MDA) levels, a marker of lipid peroxidation and oxidative injury, were markedly increased in the VAN group (Figure 1H, *p* < 0.01). Treatment with all doses of licorice and glycyrrhizic acid effectively ameliorated these VAN-induced oxidative disturbances. Notably, all treatments significantly restored GSH-Px activity (Figure 1F) and markedly attenuated the VAN-induced increase in renal MDA levels (Figure 1H). However, no significant differences in T-SOD activity were observed among the experimental groups (Figure 1G).

### 2.3. Effect of Licorice on Cell Viability, Oxidative Stress Markers and Inflammatory Markers in HK-2 Cells

**Figure 2:**
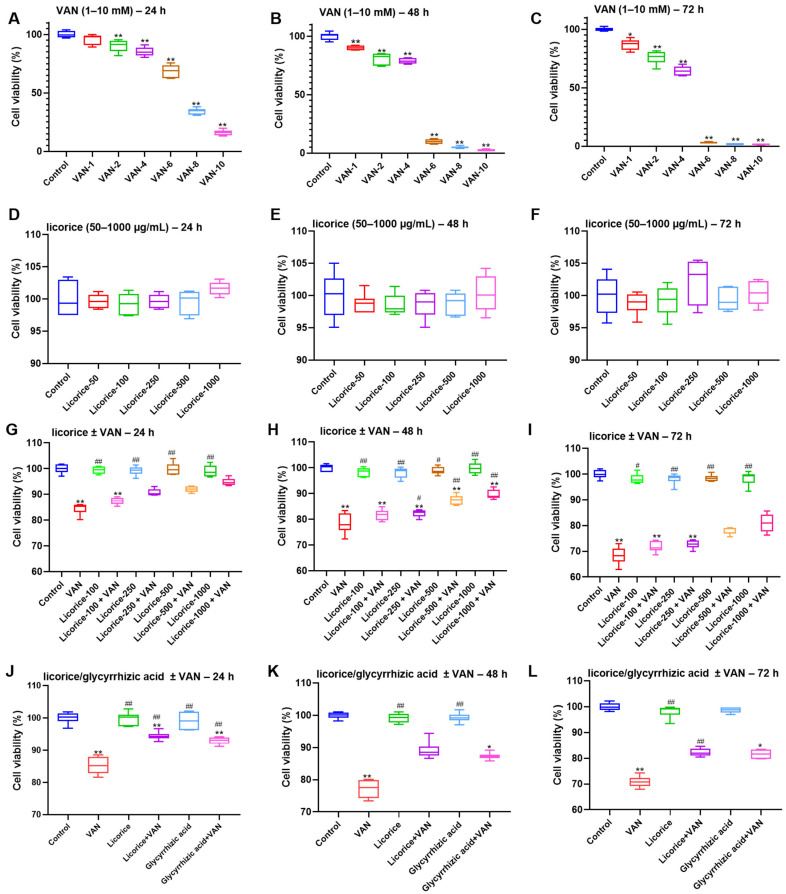
Protective effects of licorice against VAN-induced cytotoxicity in HK-2 cells (*n* = 6). (**A**–**C**) Effects of VAN (1–10 mM) on cell viability at 24, 48, and 72 h; (**D**–**F**) effects of licorice (50–1000 μg/mL) on cell viability at 24, 48, and 72 h; (**G**–**I**) Effects of licorice (100–1000 μg/mL) in the presence or absence of VAN (4 mM) on cell viability at 24, 48, and 72 h; (**J**–**L**) effects of licorice (1000 μg/mL) and glycyrrhizic acid (100 μM) in the presence or absence of VAN (4 mM) on cell viability at 24, 48, and 72 h. Data are presented as box-and-whisker plots (median, interquartile range, minimum–maximum). Statistical analysis for the data shown in (**A**,**E**,**F**,**H**,**J**) was performed using one-way ANOVA followed by Dunnett’s post hoc test. Nonparametric testing (Kruskal–Wallis H test followed by Dunn’s post hoc test) was used for the data shown in (**G**,**I**,**K**,**L**). Data shown in (**B**–**D**) were analyzed by one-way ANOVA followed by Tamhane’s T2 post hoc test. ** *p* < 0.01, * *p* < 0.05 vs. the control group; ## *p* < 0.01, # *p* < 0.05 vs. the VAN group.

As shown in Figure 2A–C, VAN reduced the viability of HK-2 cells in a dose- and time-dependent manner over the concentration range of 0–10 mM and incubation periods of 24–72 h. Based on these results, 4 mM was selected as the optimal concentration of VAN for the subsequent experiments. Cell viability assays showed that licorice at all tested concentrations (50–1000 μg/mL) exhibited no obvious cytotoxicity in HK-2 cells up to 72 h (Figure 2D–F). As shown in Figure 2G–I, licorice attenuated VAN-induced cytotoxicity in a dose- and time-dependent manner, and 1000 μg/mL was therefore selected as the optimal concentration for further study. The protective effects of licorice (1000 μg/mL) and glycyrrhizic acid (100 μM) against VAN-induced loss of cell viability were subsequently confirmed at 24, 48, and 72 h (Figure 2J–L).

**Figure 3:**
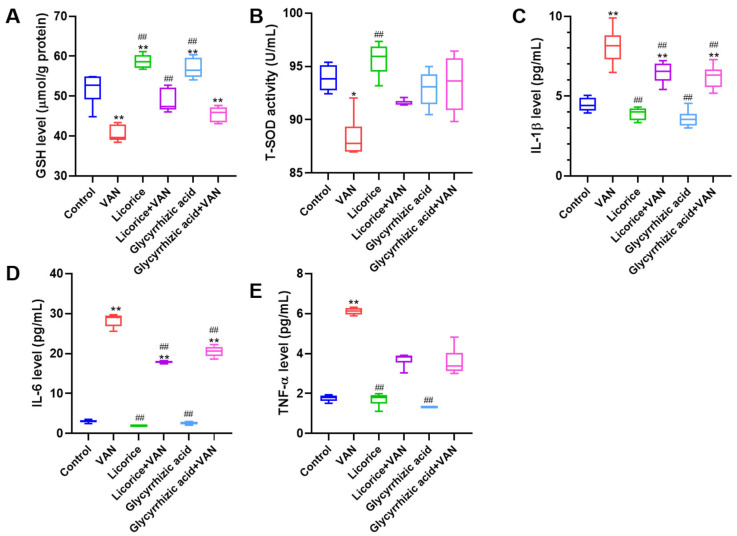
Protective effects of licorice on VAN-induced oxidative stress and inflammatory responses in HK-2 cells (*n* = 6). (**A**–**E**) Effects of licorice (1000 μg/mL) and glycyrrhizic acid (100 μM) in the presence or absence of VAN (4 mM) on GSH, T-SOD, IL-1β, IL-6, and TNF-α levels. Data are presented as box-and-whisker plots (median, interquartile range, minimum-maximum). Statistical comparisons for GSH and IL-1β levels were performed using one-way ANOVA followed by Dunnett’s post hoc test. Nonparametric testing (Kruskal–Wallis H test followed by Dunn’s post hoc test) was used for the analysis of T-SOD and TNF-α levels. IL-6 levels were analyzed by one-way ANOVA followed by Tamhane’s T2 post hoc test. ** *p* < 0.01, * *p* < 0.05 vs. the control group; ## *p* < 0.01 vs. the VAN group.

Consistent with the in vivo findings, treatment with licorice and glycyrrhizic acid effectively attenuated VAN-induced oxidative stress in HK-2 cells. VAN significantly reduced cellular GSH levels (Figure 3A, *p* < 0.01) and T-SOD activity (Figure 3B, *p* < 0.05), whereas both licorice and glycyrrhizic acid significantly restored GSH levels (*p* < 0.01). As shown in Figure 3C–E, VAN significantly increased the levels of interleukin-1β (IL-1β), interleukin-6 (IL-6), and tumor necrosis factor-α (TNF-α) in HK-2 cells compared with the control group (*p* < 0.01). Compared with the VAN group, treatment with licorice and glycyrrhizic acid significantly reversed the VAN-induced increases in IL-1β and IL-6 levels (*p* < 0.01). In addition, licorice and glycyrrhizic acid showed a trend toward reducing TNF-α levels relative to the VAN group, although the differences did not reach statistical significance.

### 2.4. Effect of Licorice on Mitochondrial Membrane Potential (MMP)

**Figure 4:**
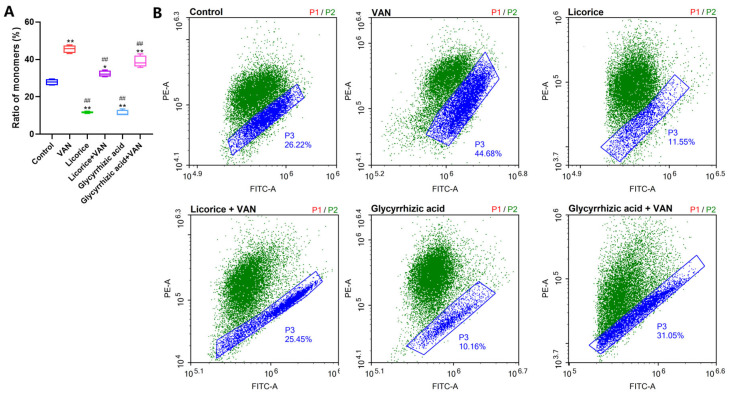
Effects of licorice and glycyrrhizic acid on VAN-induced changes in mitochondrial membrane potential (MMP) in HK-2 cells. HK-2 cells were treated with licorice (1000 μg/mL) or glycyrrhizic acid (100 μM) in the presence or absence of VAN (4 mM) for 24 h, and MMP was assessed by JC-1 staining (Ex = 585 nm, Em = 590 nm for JC-1 aggregates; Ex = 514 nm, Em = 529 nm for JC-1 monomers). (**A**) Ratio of monomers; (**B**) representative images showing the ratio of monomers in different groups. Data are presented as box-and-whisker plots (median, interquartile range, minimum-maximum). MMP levels were analyzed by one-way ANOVA followed by Dunnett’s post hoc test. ** *p* < 0.01, * *p* < 0.05 vs. the control group; ## *p* < 0.01 vs. the VAN group.

MMP is widely recognized as a sensitive indicator of mitochondrial function. Compared with the control group, the proportion of monomers was significantly increased after treatment with 4 mM VAN for 24 h (*p* < 0.01) (Figure 4), indicating that VAN induced mitochondrial membrane depolarization. Compared with the VAN group, both licorice (*p* < 0.01) and glycyrrhizic acid (*p* < 0.01) significantly reversed the VAN-induced increase in monomers, demonstrating that both treatments alleviated mitochondrial dysfunction.

### 2.5. Histological Analysis of Licorice on the Renal Tissue in Mice and Cells

**Figure 5:**
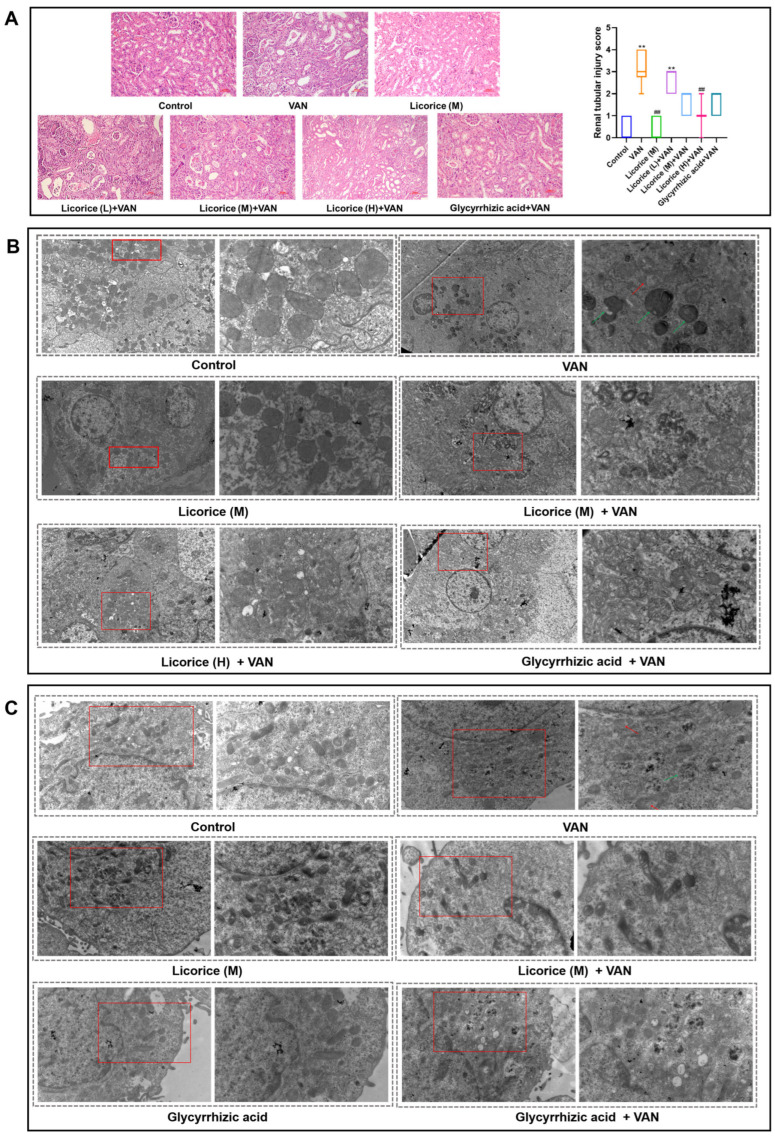
Histological and ultrastructural analysis of the protective effects of licorice on VAN-induced nephrotoxicity in mice (*n* = 10) and HK-2 cells (*n* = 4). (**A**) Hematoxylin and eosin (H&E) staining of renal tissue in mice (200× magnification); (**B**) ultrastructural changes in renal tissue examined by transmission electron microscopy (TEM) in mice (3000× and 7000× magnification); and (**C**) ultrastructural changes in HK-2 cells after treatment with licorice (1000 μg/mL) or glycyrrhizic acid (100 μM) in the presence or absence of VAN (4 mM) for 24 h, as assessed by TEM (7000× and 12,000× magnification). Red arrows indicate mitochondrial swelling, vacuolar degeneration, fragment-ed and blurred cristae; green arrows indicate autolysosomes. The doses of Licorice (L), Licorice (M), and Licorice (H) were 0.4, 2, and 10 g raw licorice/kg body weight, respectively, administered twice daily to mice. Data are presented as box-and-whisker plots (median, interquartile range, minimum-maximum). Nonparametric testing (Kruskal–Wallis H test followed by Dunn’s post hoc test) was used for the analysis of renal tubular injury scores. ** *p* < 0.01 vs. the control group; ## *p* < 0.01 vs. the VAN group.

Hematoxylin and eosin (H&E) staining and transmission electron microscopy (TEM) were used to evaluate the protective effects of licorice against VAN-induced renal injury. H&E staining revealed that the renal histology of both the control group and the Licorice (M) group was largely normal, with only occasional lymphocyte infiltration. As shown in Figure 5A, mice in the VAN group exhibited severe renal morphological damage, characterized by marked inflammatory cell infiltration, peritubular capillary dilation and congestion, vacuolar degeneration of renal tubular epithelial cells, and single-cell necrosis. Compared with the VAN group, treatment with different doses of licorice alleviated tubular vacuolar degeneration and tubular swelling, while only occasional inflammatory cell infiltration was observed in the Licorice (H) + VAN group. In the glycyrrhizic acid group, tubular epithelial cell swelling and tubular vacuolar degeneration were still present. The protective effects of licorice and glycyrrhizic acid were further supported by significant reductions in renal tubular injury scores (Figure 5A). These findings further confirm that licorice and glycyrrhizic acid can attenuate VAN-induced renal pathological injury, as demonstrated by both histopathological observations and quantitative analysis of renal tubular injury scores.

The ultrastructural changes are shown in Figure 5B. No obvious ultrastructural abnormalities were observed in the control group or the Licorice (M) group, and the mitochondria displayed clear morphology with intact cristae. In contrast, marked mitochondrial abnormalities were observed in the VAN group, including swelling, vacuolar degeneration, disruption of the double-membrane structure, fragmented and blurred cristae, and numerous abnormally enlarged autolysosomes in the cytoplasm. Licorice significantly ameliorated the VAN-induced damage to mitochondrial morphology and structure. Glycyrrhizic acid also exerted a protective effect against VAN-induced mitochondrial injury, although its effect was weaker than that observed in the Licorice (H) + VAN group. HK-2 cells were incubated with licorice (1000 μg/mL) or glycyrrhizic acid (100 μM) in the presence or absence of VAN (4 mM) for 24 h. No obvious ultrastructural abnormalities were observed in the control, licorice, or glycyrrhizic acid groups (Figure 5C). Compared with the control group, the VAN group exhibited marked destruction of the mitochondrial double-membrane and cristae structures, accompanied by occasional mitochondrial swelling and apoptotic bodies (Figure 5C). Consistent with the in vivo findings, both licorice and glycyrrhizic acid protected against VAN-induced mitochondrial structural damage in HK-2 cells. 

### 2.6. Effect of Licorice on the Intestinal Microbiota by 16S rRNA Gene Sequencing in Mice

Whether the intestinal microbiota is involved in the renoprotective effects of licorice against VAN-induced nephrotoxicity was further investigated. Venn diagrams showed that 43 operational taxonomic units (OTUs) were shared among the seven groups, while VAN treatment markedly reduced the number of OTUs compared with the control group (Figure S3A). In contrast, treatment with different doses of licorice or glycyrrhizic acid increased the number of OTUs after VAN exposure (Figure S3A). Compared with the control group, α-diversity in the Licorice (M) group was not significantly altered, whereas VAN markedly affected the Chao1 (*p* < 0.001), Shannon (*p* < 0.01), Observed species (*p* < 0.001), Faith’s PD (*p* < 0.05), and Good’s coverage (*p* < 0.05) indices (Figure S3B). For the Chao1 and Observed species indices, licorice and glycyrrhizic acid restored microbial richness, with the Licorice (M) + VAN and Licorice (H) + VAN groups showing significantly higher richness than the VAN group and approaching near-normal levels (Figure S3B). β-diversity was used to evaluate differences in the composition of the intestinal microbiota among groups. As shown by principal coordinate analysis (PCoA), the microbial composition of the control group was similar to that of the Licorice (M) group, whereas the VAN group clustered closely with the glycyrrhizic acid + VAN group. In addition, the groups receiving different doses of licorice showed similar microbial community compositions (Figure S3C). Consistently, non-metric multidimensional scaling (NMDS) analysis of microbial β-diversity demonstrated that the seven groups could be separated into distinct clusters (stress = 0.0592, Figure S3D).

**Figure 6:**
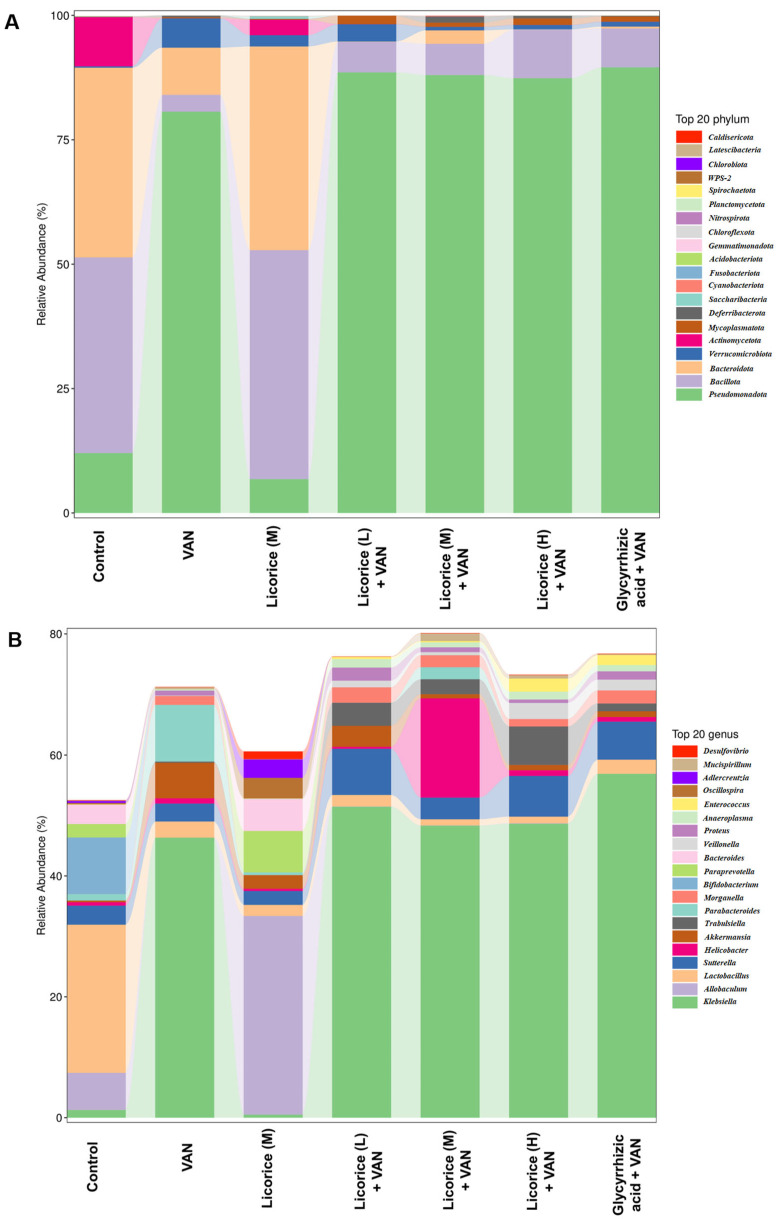
Effects of licorice on the intestinal microbiota in mice, as determined by 16S rRNA gene sequencing (*n* = 10). (**A**) Histogram of taxonomic composition at the phylum level; (**B**) histogram of taxonomic composition at the genus level. The doses of Licorice (L), Licorice (M), and Licorice (H) were 0.4, 2, and 10 g raw licorice/kg body weight, respectively, administered twice daily to mice.

To better understand the alterations in the intestinal microbiota, taxonomic compositions were further analyzed. At the phylum level, *Actinomycetota*, *Bacteroidota*, *Bacillota*, and *Pseudomonadota* accounted for more than 93% of the total microbiota (Figure 6A). In the Licorice (M) group, the relative abundances of *Bacillota* and *Bacteroidota* were decreased (Figure 6A). In contrast, the *Bacillota*/*Bacteroidota* ratio was increased in the VAN group, whereas treatment with licorice or glycyrrhizic acid reversed this change (Figure 6A). At the genus level, VAN significantly increased the relative abundances of *Parabacteroides* and *Klebsiella*, while decreasing those of *Bacteroides*, *Paraprevotella*, *Allobaculum*, and *Bifidobacterium* (Figure 6B). Licorice treatment markedly increased the abundances of *Bacteroides*, *Paraprevotella*, and *Allobaculum*, while reducing those of *Parabacteroides* and *Klebsiella* (Figure 6B). Overall, both licorice and glycyrrhizic acid reversed the VAN-induced alterations in *Parabacteroides*, *Bacteroides*, *Paraprevotella*, and *Allobaculum*. Linear discriminant analysis effect size (LEfSe) was further performed to identify differentially abundant microbial genera among the seven groups (Figure S3E). Functional prediction based on the Kyoto Encyclopedia of Genes and Genomes (KEGG) indicated that the altered microbiota was mainly associated with pathways related to carbohydrate metabolism, amino acid metabolism, metabolism of terpenoids and polyketides, energy metabolism, lipid metabolism, xenobiotics biodegradation and metabolism, and nucleotide metabolism (Figure S3F).

### 2.7. Measurements of SCFAs in the Feces

**Figure 7:**
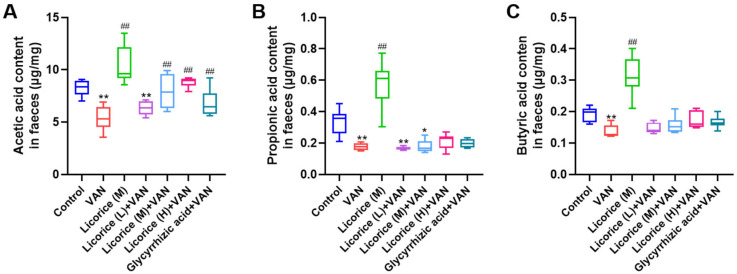
Contents of acetic acid (**A**), propionic acid (**B**), and butyric acid (**C**) in fecal samples. The doses of Licorice (L), Licorice (M), and Licorice (H) were 0.4, 2, and 10 g raw licorice/kg body weight, respectively, administered twice daily to mice. Data are presented as box-and-whisker plots (median, interquartile range, minimum–maximum). Acetic acid levels were analyzed by one-way ANOVA followed by Tamhane’s T2 post hoc test. Nonparametric testing (Kruskal–Wallis H test followed by Dunn’s post hoc test) was used for the analysis of propionic acid and butyric acid levels. ** *p* < 0.01, * *p* < 0.05 vs. the control group; ## *p* < 0.01 vs. the VAN group.

In this study, the contents of acetic acid, propionic acid, and butyric acid were measured by gas chromatography (GC). The linear correlation coefficients (*R*^2^) for acetic acid, propionic acid, and butyric acid were all greater than 0.99, indicating good linearity. The relative standard deviations (RSDs) for the stability of these three SCFAs in quality control samples were all below 15%, demonstrating satisfactory stability and reliability of the analytical method. Compared with the control group, the levels of acetic acid, propionic acid, and butyric acid were significantly decreased in the VAN group (*p* < 0.01) (Figure 7). After intervention with licorice or glycyrrhizic acid, the concentration of acetic acid was notably increased.

### 2.8. Uremic Toxins in Serum and Renal Tissue Determined by LC-MS/MS

**Figure 8:**
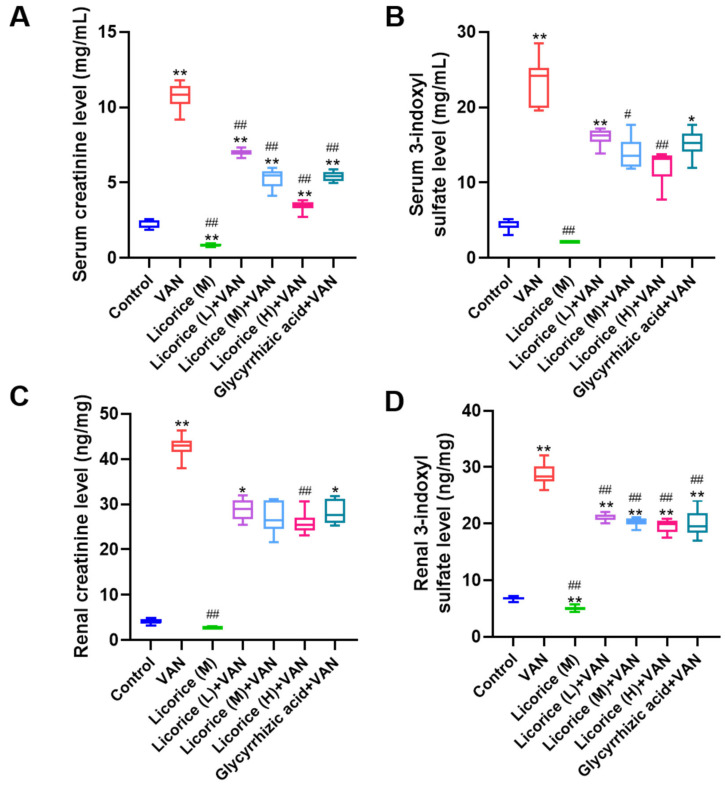
Levels of creatinine and 3-indoxyl sulfate in serum (mg/mL) and renal tissue (ng/mg). (**A**) Serum creatinine level; (**B**) Serum 3-indoxyl sulfate level; (**C**) Renal creatinine level; (**D**) Renal 3-indoxyl sulfate level. The doses of Licorice (L), Licorice (M), and Licorice (H) were 0.4, 2, and 10 g raw licorice/kg body weight, respectively, administered twice daily to mice. Data are presented as box-and-whisker plots (median, interquartile range, minimum–maximum). Serum creatinine, serum 3-indoxyl sulfate, and renal creatinine levels were analyzed by one-way ANOVA followed by Tamhane’s T2 post hoc test. Nonparametric testing (Kruskal–Wallis H test followed by Dunn’s post hoc test) was used for the analysis of renal 3-indoxyl sulfate levels. ** *p* < 0.01, * *p* < 0.05 vs. the control group; ## *p* < 0.01, # *p* < 0.05 vs. the VAN group.

In this study, the levels of uremic toxins in serum and renal tissue were quantified by LC-MS/MS using a previously established method in our laboratory [30], which demonstrated satisfactory linearity, accuracy, and precision. Compared with the control group, the serum levels of creatinine, 3-indoxyl sulfate, hippuric acid, 3-(3,4-dihydroxyphenyl)-L-alanine, 1-methylinosine, and N2,N2-dimethylguanosine were significantly elevated in the VAN group (*p* < 0.01) (Figure 8 and Figure S4). Treatment with licorice and glycyrrhizic acid reduced the VAN-induced increases in the serum levels of creatinine, 3-indoxyl sulfate, hippuric acid, and N2,N2-dimethylguanosine (Figure 8 and Figure S4). Similarly, compared with the control group, renal levels of creatinine, 3-indoxyl sulfate, phenylacetyl-L-glutamine, indole-3-acetic acid, hippuric acid, 3-(3,4-dihydroxyphenyl)-L-alanine, and 1-methylinosine were markedly increased in the VAN group (*p* < 0.01) (Figure 8 and Figure S4). Licorice and glycyrrhizic acid treatment attenuated the VAN-induced elevations in renal creatinine, 3-indoxyl sulfate, phenylacetyl-L-glutamine, and indole-3-acetic acid levels (Figure 8 and Figure S4).

## 3. Discussion

### 3.1. Quality Control of Licorice Aqueous Extract

The contents of glycyrrhizic acid and liquiritin in raw licorice, as determined by HPLC, were 2.79 ± 0.36% and 0.66 ± 0.13%, respectively, both of which exceeded the requirements of the Pharmacopoeia of the People’s Republic of China (2020 and 2025 editions) [31,32]. In the LC-MS/MS analysis, six major constituents of licorice, namely liquiritin, isoliquiritigenin, quercetin, liquiritin apioside, glycyrrhizic acid, and glycyrrhetinic acid, were selected for qualitative and quantitative evaluation. However, after optimization of the mobile phase composition, the responses of glycyrrhizic acid and glycyrrhetinic acid remained too weak for reliable quantitative analysis.

### 3.2. Licorice Improves VAN-Induced Nephrotoxicity in Mice

The renoprotective effects of licorice against VAN-induced nephrotoxicity were confirmed in the present study. According to the Pharmacopoeia of the People’s Republic of China (2020 and 2025 editions) [31,32], the recommended daily dose of raw licorice for humans is 2–10 g. Accordingly, 10 g was used as the basis for calculating the equivalent dose in mice, yielding a medium dose of 2 g/kg for Licorice (M) based on the body surface area conversion formula between humans and mice. The low dose (0.4 g/kg, Licorice L) was set at one-fifth of the medium dose, whereas the high dose (10 g/kg, Licorice H) was set at five times the medium dose. This dosing strategy is commonly used in pharmacological studies to evaluate dose-effect relationships [33]. Moreover, this dose range is consistent with the safe range reported in previous mouse studies of licorice [34]. In this study, licorice reversed VAN-induced changes in kidney index, serum creatinine, renal oxidative stress markers, and renal Kim-1 levels. VAN exposure significantly decreased renal GSH-Px activity and increased renal MDA levels (Figure 1F–H). Licorice markedly restored renal GSH-Px activity while reducing MDA levels, suggesting that its antioxidant activity may play a key role in protecting against nephrotoxicity (Figure 1). These findings are consistent with previous in vivo studies demonstrating the antioxidant effects of licorice [27,35]. Similar protective effects were also observed in HK-2 cells. Based on the HPLC quantification of glycyrrhizic acid in licorice, 500 μg/mL licorice corresponded to approximately 50 μM glycyrrhizic acid. In the present study, relatively high concentrations were used to evaluate the renoprotective effects of licorice and glycyrrhizic acid against VAN, and comparable or even higher concentrations have also been reported to exert renoprotective activity [36,37]. In addition, licorice and glycyrrhizic acid improved cell viability (Figure 2) and restored GSH and T-SOD levels in HK-2 cells (Figure 3A,B). VAN significantly increased the levels of inflammatory markers, consistent with previous reports [38]. Furthermore, licorice and glycyrrhizic acid reversed the VAN-induced increases in IL-1β, IL-6, and TNF-α in vitro (Figure 3C–E). Histopathological changes induced by VAN, as shown by H&E staining, were consistent with those reported in a previous study [39]. Both licorice and glycyrrhizic acid alleviated VAN-induced nephrotoxicity, as evidenced by improved histological features (Figure 5A).

### 3.3. Licorice Improves the Mitochondrial Function and Structure

Compared with the control group, the ratio of JC-1 monomers was markedly increased in the VAN group in vitro, indicating mitochondrial membrane depolarization. Both licorice and glycyrrhizic acid significantly reversed this change (Figure 4), suggesting that they alleviated VAN-induced mitochondrial dysfunction. Mitochondrial ultrastructure was then further evaluated. The mitochondrial structural damage induced by VAN was consistent with that reported in a previous study [39]. Both the in vivo and in vitro observations of mitochondrial morphology further confirmed the protective effects of licorice against VAN-induced mitochondrial injury (Figure 5B,C).

### 3.4. Effect of Licorice on the Intestinal Microbiota

Compared with the control group, treatment with different doses of licorice and glycyrrhizic acid reversed the VAN-induced reduction in OTU numbers (Figure S3A), although no statistically significant differences in α-diversity were observed (Figure S3B), which is consistent with a previous study [27]. Both licorice and glycyrrhizic acid also reversed the VAN-induced increase in the *Bacillota*/*Bacteroidota* ratio (Figure 6A). At the genus level, *Bacteroides*, a SCFA-producing bacterium, showed increased relative abundance in the Licorice (M), Licorice (M) + VAN, and Glycyrrhizic acid + VAN groups (Figure 6B). Interestingly, *Bacteroides* abundance has also been reported to be decreased in hyperuricemic nephropathy [40,41], diabetic kidney disease [42], and cisplatin-induced AKI [43]. Moreover, several renoprotective interventions have been shown to alleviate kidney injury by increasing the relative abundance of *Bacteroides* [40,41,42,43]. Similarly, *Paraprevotella* and *Allobaculum* were significantly decreased in the VAN group, whereas licorice and glycyrrhizic acid increased their relative abundances. Yu, M. et al. [44] reported that *Paraprevotella* was positively correlated with estimated glomerular filtration rate in patients with antineutrophil cytoplasmic antibody-associated vasculitis and kidney injury. *Allobaculum* was also found to be relatively more abundant in the high-dose San-Huang-Yi-Shen capsule group than in the diabetic nephropathy group [42]. In contrast, *Parabacteroides* was significantly increased in the VAN group, whereas licorice and glycyrrhizic acid reduced its abundance. Similarly, *Parabacteroides* was markedly elevated in rats with hyperuricemic nephropathy, and this alteration was improved by Astragaloside IV treatment [45]. Zhang, L. et al. [46] also found that *Parabacteroides* was significantly enriched in both early- and late-stage diabetic kidney disease patients. Taken together, these findings suggest that licorice and glycyrrhizic acid may exert renoprotective effects against VAN-induced nephrotoxicity by modulating the abundances of *Bacteroides*, *Paraprevotella*, *Allobaculum*, and *Parabacteroides*. It has been well established that intestinal microbiota dysbiosis and AKI interact bidirectionally: AKI can induce gut dysfunction, whereas dysbiosis can in turn aggravate AKI progression [47].

### 3.5. Effect of Licorice on SCFAs in the Feces

Several recent studies have shown that SCFAs play an important role in AKI, and impaired SCFA production may lead to the loss of anti-inflammatory and antioxidant protection [47]. In the present study, the levels of acetic acid, propionic acid, and butyric acid were significantly decreased in the VAN group, whereas licorice and glycyrrhizic acid increased the level of acetic acid (Figure 7). These changes were consistent with the alterations observed in SCFA-producing microbiota, particularly *Bacteroides*, which is known to produce acetic acid and propionic acid [48]. A growing number of studies have demonstrated the therapeutic potential of TCMs in the treatment of AKI. For example, Zou, Y.T. et al. [49] reported that Qiong-Yu-Gao significantly alleviated gut dysbiosis and increased SCFA levels, including acetic acid and butyric acid. Moreover, SCFAs themselves have been reported to prevent renal injury and protect kidney function in both experimental and clinical settings [50,51,52,53].

### 3.6. Effect of Uremic Toxins in Serum and Renal Tissue

Gut microbiota dysbiosis can promote the accumulation of certain uremic toxins, such as p-cresyl sulfate, thereby enhancing oxidative stress and inflammation and ultimately contributing to tissue injury [47]. In addition, dysregulation of p-cresyl sulfate, indoxyl sulfate, and indole-3-acetic acid has been implicated in renal injury in both AKI and chronic kidney disease through activation of aryl hydrocarbon receptor signaling and the promotion of inflammatory and fibrotic processes [54]. In the present study, multiple uremic toxins were altered in both serum and renal tissue (Figure 8 and Figure S4), further demonstrating the impact of VAN on uremic toxin metabolism and accumulation. Licorice and glycyrrhizic acid reduced the accumulation of several of these uremic toxins. Previous studies have also reported that VAN can increase indoxyl sulfate levels [39,55]. Moreover, a number of studies have shown that TCMs exert nephroprotective effects by modulating the gut microbiota and its metabolites, including SCFAs and uremic toxins, thereby reducing oxidative stress and inflammation [56,57,58]. For example, Zou, Y.T. et al. [49] reported that Qiong-Yu-Gao significantly alleviated gut dysbiosis and reduced uremic toxin accumulation in cisplatin-induced AKI.

### 3.7. The Potential Protective Mechanism of Licorice and Its Compositions

The renoprotective effects and underlying mechanisms of licorice and its constituents against nephrotoxicity have been reported in several previous studies. Licorice extracts have been shown to significantly attenuate brucine-induced nephrotoxicity through suppression of oxidative stress, inhibition of the mitochondria-mediated apoptotic pathway, and modulation of signal transducer and activator of transcription 3 (STAT3) activation [35]. Glycyrrhizic acid has also been reported to protect against tacrolimus-induced kidney injury by improving lysosomal function and regulating autophagy [36]. In addition, its protective effects against cisplatin-induced nephrotoxicity may be associated with upregulation of Nrf2 and downregulation of nuclear factor-κB (NF-κB) in the kidney [59]. Furthermore, glycyrrhizic acid alleviated sepsis-induced renal injury in vivo by reducing lipopolysaccharide-induced oxidative stress, modulating extracellular signal-regulated kinase signaling, inhibiting NF-κB activation, and suppressing apoptosis-related protein expression [60]. Isoliquiritigenin has likewise been shown to protect against cisplatin-induced nephrotoxicity in cells, at least in part through induction of heme oxygenase-1 expression, which may be associated with Nrf2 nuclear translocation [61]. In addition, isoliquiritigenin ameliorated lipopolysaccharide-induced AKI by suppressing NF-κB p65 nuclear translocation and inhibiting inflammatory responses [62].

In this study, licorice exhibited protective effects against VAN-induced nephrotoxicity, which may be associated with its antioxidant, anti-inflammatory, and mitochondria-protective activities, as well as its regulation of the intestinal microbiota, SCFAs, and uremic toxins. Notably, this study has several limitations. First, the upstream signaling pathways potentially involved in the renoprotective effects of licorice, including KEAP1, Nrf2, NF-κB, and NF-κB p65, were not directly validated by Western blot analysis. Second, the long-term safety and efficacy of licorice were not evaluated. Third, it remains unclear whether the renoprotective effects of licorice are primarily attributable to its direct antioxidant activity or to its modulation of the intestinal microbiota. Future studies should therefore focus on the following aspects: (1) elucidating the precise molecular mechanisms underlying the protective effects of licorice against VAN-induced nephrotoxicity; (2) conducting more detailed dose–response and time-course studies, including comparisons among pre-treatment, co-treatment, and post-treatment regimens; (3) evaluating the long-term safety and efficacy of licorice; and (4) clarifying the causal relationship between alterations in the intestinal microbiota and renoprotection through fecal microbiota transplantation or germ-free animal models.

## 4. Materials and Methods

### 4.1. Chemicals and Reagents

VAN was purchased from Aladdin Scientific Corporation (Shanghai, China). Licorice (dried roots of *Glycyrrhiza uralensis* Fisch.) was obtained from Guanlan Chinese Medicine Group Company (Lanzhou, China). Glycyrrhizic acid, liquiritin and isoliquiritigenin were purchased from Shanghai Rhawn Chemical Technology Co., Ltd. (Shanghai, China). Quercetin, liquiritin apioside and glycyrrhetinic acid were purchased from Wuhan ChemFaces Biochemical Co., Ltd. (Wuhan, China). Creatinine, GSH, GSH-Px, T-SOD and MDA assay kits were purchased from Nanjing Jiancheng Bioengineering Institute (Nanjing, China). The mouse Kim-1 ELISA assay kit was supplied by Shanghai Enzyme-linked Biotechnology Corporation (Shanghai, China). ELISA kits for IL-6, IL-1β, and TNF-α were purchased from Ruixin Biotech (Quanzhou, China). A 2.5% glutaraldehyde solution was purchased from Solarbio Science & Technology Co., Ltd. (Beijing, China). All other reagents were of analytical grade or higher, and deionized water was used throughout the experiments.

### 4.2. Preparation and Quality Control of Licorice Aqueous Extract

Licorice (dried roots of *Glycyrrhiza uralensis* Fisch.) was authenticated by Prof. Jianyin Li (School of Pharmacy, Lanzhou University, Lanzhou, China) and a voucher specimen (NO. 621125LY0314) has been deposited at Herbarium of Lanzhou University (Traditional Chinese Medicine). The licorice was extracted independently in triplicate. Briefly, 200 g of licorice was decocted twice in 2000 mL of boiling water for 1 h each time. The combined decoctions were filtered and concentrated under reduced pressure at 50 °C. According to the Pharmacopoeia of the People’s Republic of China (2020 edition) [31], high-performance liquid chromatography with ultraviolet detection (HPLC-UV) was used to detect glycyrrhizic acid, liquiritin and isoliquiritigenin. The chromatographic separation was performed on a Sunfire C18 column (4.6 mm × 250 mm, 5 μm; Waters Corporation, Milford, MA, USA). The mobile phase consisted of acetonitrile (A) and 0.05% phosphoric acid aqueous solution (B), using the following gradient elution program: 19% A (0–8 min), 19–50% A (8–35 min), 50–100% A (35–36 min), and 100–19% A (36–40 min). The detection wavelength was set at 237 nm, and the column temperature was maintained at 40 °C.

An AB SCIEX Triple Quad™ 4500 mass spectrometer (AB Sciex, Framingham, MA, USA) was used for the LC-MS/MS analysis of four compounds in the aqueous extract of licorice. Chromatographic separation was performed on a ZORBAX RR Stable Bond C18 analytical column (Agilent 861953-902, 4.6 mm × 100 mm, 3.5 µm) (Agilent Technologies, Inc., Santa Clara, CA, USA) maintained at 40 °C. The mobile phase consisted of water and methanol (20:80, *v*/*v*), delivered at a flow rate of 0.5 mL/min, with an injection volume was 2 μL. For mass spectrometry, negative electrospray ionization (ESI) in multiple reaction monitoring (MRM) mode detection was employed. The mass spectrometric parameters were as follows: ion source temperature, 550 °C; ion spray voltage, 5500 V; curtain gas (nitrogen), 30 psi; nebulizer gas (GS1), 50 psi; and auxiliary gas (GS2), 50 psi. The ion pairs, declustering potential, collision energy, collision cell exit potential are shown in Table S1.

### 4.3. Animals

Six-week-old male wild-type C57BL/6 mice used in this study were obtained from the Animal Center of Lanzhou Institute of Biological Products Co., Ltd. (Lanzhou, China). All animal experiments were conducted in accordance with the National Institutes of Health Guide for the Care and Use of Laboratory Animals and were approved by the Ethics Committee of The First Hospital of Lanzhou University (NO. LDYYLL2019-141). Before treatment, the mice were housed under standard laboratory conditions (22 ± 2 °C, 12 h light/12 h dark cycle) with free access to food and water.

### 4.4. Cell Culture

The human proximal tubular epithelial cells (HK-2 cells) were obtained from the Institute of Basic Medical Sciences, Chinese Academy of Medical Science (Beijing, China). The cells were cultured in minimum essential medium (MEM, HyClone, Cytiva, Logan, UT, USA) media supplemented with 10% (*v*/*v*) fetal bovine serum (Gibco, Grand Island, NY, USA) and 0.1% (*v*/*v*) penicillin–streptomycin (Gibco, Grand Island, NY, USA), and maintained at 37 °C in a humidified atmosphere containing 5% CO_2_.

### 4.5. Effect of Licorice on Nephrotoxicity of VAN in Mice

C57BL/6 mice were randomly assigned to seven groups (*n* = 10 per group), as summarized in Table 1. The doses of Licorice (L), Licorice (M), and Licorice (H) were 0.4, 2, and 10 g raw licorice/kg body weight, respectively, administered twice daily, corresponding to 54, 270, and 1350 mg/kg of licorice aqueous extract in mice. Glycyrrhizic acid, the major active constituent and quality control of licorice in Pharmacopoeia of the People’s Republic of China (2020 and 2025 editions) [31,32], was included as a separate treatment group to further evaluate the renoprotective effects of licorice against VAN-induced nephrotoxicity. The dose of glycyrrhizic acid in the Glycyrrhizic acid + VAN group was matched to its content in the Licorice (M) group, corresponding to 50 mg/kg in mice. VAN was administered once daily by intraperitoneal (*i.p.*) injection for 7 consecutive days to establish a nephrotoxicity model, as previously described [63]. Twelve hours after the last administration, the mice were fasted and then humanely euthanized. Blood samples were collected from the orbital sinus, and fecal samples were collected from the colon. Renal tissues were subsequently harvested for histological evaluation, ultrastructural observation of mitochondria, and measurement of oxidative stress markers.

### 4.6. Effect of Licorice on Cell Viability Determined by MTT Assay in HK-2 Cells

Cell viability was determined using the MTT assay as previously described [64]. HK-2 cells were seeded in 96-well plates and cultured for 24 h in MEM supplemented with 10% FBS and 0.1% penicillin-streptomycin. The culture medium was then removed, and the cells were exposed to different treatments for 24, 48, or 72 h. After treatment, the medium was discarded, and fresh MEM (900 μL) and MTT solution (5 mg/mL, 100 μL) were added to each well, followed by incubation at 37 °C for 4 h. The supernatant was subsequently removed, and the resulting formazan crystals were dissolved in DMSO (150 μL). Absorbance was then measured at 570 nm.

Briefly, HK-2 cells were treated with MEM containing VAN at concentrations of 0, 2, 4, 6, 8, or 10 mM to determine the optimal VAN concentration. The cells were then incubated with licorice aqueous extract at 50, 100, 250, 500, or 1000 μg/mL to evaluate its potential cytotoxicity. Subsequently, the optimal renoprotective concentration of licorice aqueous extract against VAN-induced injury was determined. Finally, the cytoprotective effects of licorice aqueous extract (100, 250, 500, or 1000 μg/mL) and glycyrrhizic acid against VAN-induced cytotoxicity were assessed. The dose of glycyrrhizic acid in the Glycyrrhizic acid + VAN group was matched to the glycyrrhizic acid content present in 1000 μg/mL licorice aqueous extract, corresponding to 100 μM in the cell culture system.

### 4.7. Detection of Creatinine, Kim-1, Oxidative Stress Markers and Inflammatory Markers

Serum creatinine, renal oxidative stress markers, including GSH, GSH-Px, and T-SOD, renal MDA levels, as well as GSH and T-SOD levels in the HK-2 cell culture supernatant, were measured using commercial assay kits according to the manufacturers’ instructions (Nanjing Jiancheng Bioengineering Institute, Nanjing, China) [8]. Mouse Kim-1 levels were determined using a commercial kit in accordance with the manufacturer’s instructions [64]. The levels of IL-6, IL-1β, and TNF-α in the HK-2 cell culture supernatant were quantified using ELISA kits (Ruixin Biotech, Quanzhou, China) according to the manufacturers’ instructions [65].

### 4.8. Effect of Licorice on Mitochondrial Membrane Potential (MMP)

The MMP was measured using JC-1 (5,5′,6,6′-tetrachloro- 1,1′,3,3′-tetraethylbenzimidazolylcarbocyanine iodide) (MedChemExpress, Monmouth Junction, NJ, USA) according to the manufacturer’s instructions. The procedure for MMP detection was performed as previously described [66]. HK-2 cells were treated with VAN (4 mM), licorice in the presence or absence of VAN, or glycyrrhizic acid in the presence or absence of VAN for 24 h. The culture medium was then replaced with JC-1 working solution (2 μM), and the cells were incubated at 37 °C for 20 min. After washing three times with PBS, fluorescence intensity was analyzed by flow cytometry (BD Biosciences, San Jose, CA, USA). JC-1 aggregates and monomers were detected in the PE channel (565–605 nm) and FITC channel (515–545 nm), respectively.

### 4.9. Hematoxylin and Eosin (H&E) Staining

In brief, renal tissues from mice were fixed for 24 h and stained with hematoxylin and eosin (H&E) as previously described [67]. The prepared tissue sections were examined and photographed under a light microscope for histopathological evaluation. The extent of tubular injury was assessed semi-quantitatively by two pathologists. Ten random fields were selected from each renal tissue section in each group. Tubular injury was evaluated using a five-point scoring system as follows: 0, no lesions; 1, mild injury (≤25%); 2, moderate injury (>25–50%); 3, severe injury (>50–75%); and 4, very severe injury (>75% to <100%) [68].

### 4.10. Transmission Electron Microscopy (TEM)

Fresh renal tissue samples (≤1 mm^3^) or HK-2 cells were fixed in 2.5% glutaraldehyde at 4 °C. Sample processing was performed as previously described by Zhang, M. et al. [67]. The specimens were then examined using a field-emission high-resolution transmission electron microscope (HRTEM; Tecnai G2 F30) (FEI Corporation, Hillsboro, OR, USA). Images were finally acquired with a CCD camera (Gatan 894 Ultrascan 1000) (Gatan Corporation, Pleasanton, CA, USA).

### 4.11. Effect of Licorice on the Intestinal Microbiota by 16S rRNA Gene Sequencing in Mice

Mouse fecal samples were collected and stored at −80 °C until analysis. 16S rRNA gene sequencing was performed to characterize the intestinal microbiota. DNA extraction and sequencing were conducted Metabo-Profile Biotechnology Co., Ltd (Shanghai, China). The sequencing workflow mainly consisted of the following steps:

(1) DNA extraction: Microbial DNA was extracted from fecal samples according to the manufacturer’s instructions. DNA concentration and quality were assessed by 1.2% agarose gel electrophoresis.
(2) PCR amplification: The V3-V4 region of the bacterial 16S ribosomal RNA gene was amplified using barcoded universal primers, 341F (5′-CCTACGGGRSGCAGCAG-3′) and 806R (5′-GGACTACVVGGGTATCTAATC-3′). To obtain clean sequencing templates, PCR amplicons were purified using VAHTS DNA Clean Beads (Vazyme International LLC., Nanjing, China).
(3) Quantification of PCR products: PCR products were quantified using the Quant-iT PicoGreen dsDNA Assay Kit (Thermo Fisher Scientific, Waltham, MA, USA) and a microplate reader (BioTek FLx800) (Waltham, MA, USA).
(4) Library preparation and sequencing: Libraries were prepared using the TruSeq Nano DNA LT Library Prep Kit (Illumina, Inc., San Diego, CA, USA). Purified products were quantified with the Promega QuantiFluor system (Promega Corporation, Beijing, China). Qualified libraries were sequenced on a NovaSeq 6000 platform using the No-vaSeq 6000 SP Reagent Kit (500 cycles) (Illumina, Inc., San Diego, CA, USA).

For bioinformatic analysis, sequences with 97% similarity were clustered into operational taxonomic units (OTUs). Alpha and beta diversity were calculated using QIIME2 (version 2019.4). Principal coordinate analysis (PCoA) and non-metric multidimensional scaling (NMDS) were performed and visualized using QIIME2 and R software (version 3.4.4). Permutational multivariate analysis of variance (PERMANOVA) was used to evaluate the significance of clustering patterns. Differential microbial features were identified using linear discriminant analysis effect size (LEfSe). Functional prediction was performed using Phylogenetic Investigation of Communities by Reconstruction of Unobserved States (PICRUSt) in combination with the Kyoto Encyclopedia of Genes and Genomes (KEGG) pathway database.

### 4.12. Measurements of SCFAs in the Feces

Reference solutions and fecal samples were prepared as previously described by Zou, Y.T. et al. [49]. Acetic acid, propionic acid, and butyric acid were separated on an Agilent HP-FFAP column (30 m × 530 μm, 1 μm) (Agilent, Santa Clara, CA, USA).and quantified using an Agilent 7890B gas chromatograph coupled with a flame ionization detector (GC-FID; Agilent, Santa Clara, CA, USA). 2-Methylvaleric acid was used as the internal standard, and SCFA levels were expressed as the amount per gram of feces. The GC conditions were as follows: the initial oven temperature was set at 60 °C and maintained for 3 min, then increased to 200 °C at 10 °C/min, followed by a further increase to 225 °C at 30 °C/min and held for 3 min. Nitrogen was used as the carrier gas at a flow rate of 1.0 mL/min. The injector temperature was 230 °C with a split ratio of 5:1. The FID temperature was set at 250 °C, with a hydrogen flow rate of 30 mL/min and an air flow rate of 400 mL/min. The injection volume was 1 μL.

### 4.13. Uremic Toxins in Serum and Renal Tissue Determined by LC-MS/MS

Samples (50 μL) were mixed with 50 μL of internal standard solution (d5-hippuric acid for negative ESI mode and d3-creatinine for positive ESI mode) and 100 μL of acetonitrile for protein precipitation. The mixtures were then vortexed and centrifuged, and 10 μL of the resulting supernatant was subjected to LC-MS/MS analysis.

The concentrations of uremic toxins were determined by LC-MS/MS using an Agilent 1260 liquid chromatography system coupled with a 6460 triple-quadrupole mass spectrometer (Agilent Technologies, Santa Clara, CA, USA). The analytical conditions were based on a previously established method developed in our laboratory [30], which showed satisfactory linearity, accuracy, and precision. Chromatographic separation was performed on a ZORBAX RR Stable Bond C18 analytical column (Agilent 861953-902, 4.6 mm × 100 mm, 3.5 µm) (Agilent Technologies, Inc., Santa Clara, CA, USA) maintained at 30 °C. For positive ESI mode, the mobile phase consisted of water containing 0.1% formic acid and methanol (60:40, *v*/*v*) delivered at a flow rate of 0.65 mL/min. For negative ESI mode, the mobile phase consisted of water and acetonitrile (60:40, *v*/*v*) at the same flow rate. The mass spectrometric parameters for the uremic toxins are listed in Table S2.

### 4.14. Data Processing and Statistical Analysis

Data visualization was performed using GraphPad Prism 8.0.1 software (GraphPad, La Jolla, CA, USA). All data are presented as box-and-whisker plots (median, interquartile range, minimum-maximum). Statistical analyses were conducted using SPSS 27.0 software (SPSS Inc., Chicago, IL, USA). Normality was assessed using the Shapiro–Wilk test. For normally distributed data, one-way analysis of variance (ANOVA) was performed, followed by Levene’s test for homogeneity of variance. Dunnett’s post hoc test was used for multiple comparisons when variances were homogeneous, whereas Tamhane’s T2 post hoc test was applied when variances were unequal. For non-normally distributed data, the Kruskal–Wallis H test was used, followed by Dunn’s post hoc test with Bonferroni correction for pairwise comparisons. A *p* value < 0.05 was considered statistically significant.

## 5. Conclusions

In conclusion, licorice exerted protective effects against VAN-induced nephrotoxicity both in vivo and in vitro, suggesting the involvement of multiple mechanisms, including modulation of oxidative stress, mitochondrial structure and function, inflammation, the intestinal microbiota-SCFA axis, and uremic toxins.

## Figures and Tables

**Table 1:** Groups and treatment (*n* = 10).

Groups	Treatment (For 7 Days) Intragastric Administration (*p.o.*)	Treatment (For 7 Days) Intraperitoneal Injection (*i.p.*)
Control	water (0.2 mL/10 g, twice daily)	saline (0.2 mL/10 g, once daily)
VAN	water (0.2 mL/10 g, twice daily)	VAN (600 mg/kg, once daily)
Licorice(M)	licorice aqueous extract (270 mg/kg, twice daily)	saline (0.2 mL/10 g, once daily)
Licorice(L) + VAN	licorice aqueous extract (54 mg/kg, twice daily)	VAN (600 mg/kg, once daily)
Licorice(M) + VAN	licorice aqueous extract (270 mg/kg, twice daily)	VAN (600 mg/kg, once daily)
Licorice(H) + VAN	licorice aqueous extract (1350 mg/kg, twice daily)	VAN (600 mg/kg, once daily)
Glycyrrhizic acid + VAN	glycyrrhizic acid (50 mg/kg, twice daily)	VAN (600 mg/kg, once daily)

## Data Availability

The original contributions presented in this study are included in the article (and Supplementary Materials). Further inquiries can be directed to the corresponding author(s).

## Supplementary Materials

The following supporting information can be downloaded at: https://www.mdpi.com/article/10.3390/ph19050728/s1, Figure S1: Chromatograms of reference substance (A) and test samples (B). Note: 1. liquiritin, 2. glycyrrhizic acid, 3. Isoliquiritigenin; Figure S2: Chromatograms and mass spectrometry of compounds. (A) Chromatograms of liquiritin, isoliquiritigenin, quercetin and liquiritin apioside, respectively; (B) Chromatograms of mixture of liquiritin, isoliquiritigenin, quercetin and liquiritin apioside; (C) Mass spectrometry of compounds; Figure S3: Effect of licorice on the intestinal flora by 16s rDNA sequencing in mice (n = 10). (A) The Venn (ASV/OTUs); (B) The microbial α-diversity in seven groups; (C) The microbial β diversity calculated by PCoA; (D) The microbial β diversity calculated by NMDS; (E) LDA effect size (LEfSe) among seven groups; (F) The KEGG pathways. The dosages of Licorice (L), Licorice (M) and Licorice (H) were set at 0.4, 2 and 10 g (raw licorice)/(kg body weight) twice daily to mice; Figure S4: The levels of uremic toxins in serum and in renal tissue. (A) Serum 3-(3,4-dihydroxyphenyl)-L-alanine (mg/mL); (B) Serum 1-methyl-inosine (ng/mL); (C) Serum N2, N2-Dimethylguanosine (ng/mL); (D) Serum N-acetylcytidine (ng/mL); (E) Serum Hippuric acid (ng/mL); (F) Renal phenylacetyl-L-glutamine (ng/mg); (G) Renal Indole-3-acetic acid (ng/mg); (H) Renal 3-(3,4-dihydroxyphenyl)-L-alanine level (ng/mg); (I) Renal 1-methyl-inosine (ng/mg); (J) Renal N2, N2-Dimethylguanosine (ng/mg); (K) Renal N-acetylcytidine (ng/mg); (L) Renal Hippuric acid (ng/mg). The dosages of Licorice (L), Licorice (M) and Licorice (H) were set at 0.4, 2 and 10 g (raw licorice)/(kg body weight) twice daily to mice. All data were presented as box-and-whisker plots (median, interquartile range, min-max). Non-parametric tests (Kruskal Wallis H test with Dunn’s post hoc test) were used for statistical analysis of the data presented in Figure S4 (C,D,F,G,J,K,L). Data shown in Figure S4 (A,B,E,H,I) were analyzed by one way ANOVA followed by Tamhane’s T2 post hoc test. ** *p* < 0.01, * *p* < 0.05 vs. the control group; ## *p* < 0.01, # *p* < 0.05 vs. the VAN group; Table S1: Mass spectrometry detection conditions for four compounds; Table S2: Mass spectrometry detection conditions for different uremic toxins.

## Abbreviations

The following abbreviations are used in this manuscript:

AKI	Acute kidney injury
ESI	Electrospray ionization
FID	Flame Ionization Detector
GC	Gas chromatograph
GSH	Glutathione
GSH-Px	Glutathione peroxidase
H&E	Hematoxylin and Eosin
HPLC	High performance liquid chromatography
IL-1β	Interleukin-1β
IL-6	Interleukin-6
KEAP1	Kelch-like ECH-associated protein 1
KEGG	Kyoto encyclopedia of genes and genomes
LC-MS/MS	Liquid chromatography-tandem mass spectrometry
LEfSe	Linear discriminant analysis effect size
MDA	Malondialdehyde
MEM	Minimum essential medium
MMP	Mitochondrial membrane potential
NMDS	Nonmetric multidimensional scaling
Nrf2	Nuclear factor (erythroid-derived 2)-related factor 2
OTUs	Operational taxonomic units
PCoA	Principal coordinate analysis
SCFAs	Short chain fatty acids
TCMs	Traditional Chinese medicines
TNF-α	Tumor necrosis factor-α
T-SOD	Total superoxide dismutase
VAN	Vancomycin
