# Supplementary material for: Protective Effects of Licorice (Glycyrrhiza uralensis) Against Vancomycin-Induced Nephrotoxicity In Vivo and In Vitro
Source: Pharmaceuticals (Basel). 2026 May 4;19(5):728. doi: 10.3390/ph19050728 (PMC13209765; doi:10.3390/ph19050728)
Supplement: Supplementary file 1 [file pharmaceuticals-19-00728-s001.zip › Legends of Figures.pdf]

Figure S1. Chromatograms of reference substance (A) and test samples (B).
Note: 1. liquiritin, 2. glycyrrhizic acid, 3. Isoliquiritigenin

Figure S2. Chromatograms and mass spectrometry of compounds. (A) Chromatograms of liquiritin, isoliquiritigenin, quercetin and liquiritin apioside, respectively; (B) Chromatograms of mixture of liquiritin, isoliquiritigenin, quercetin and liquiritin apioside; (C) Mass spectrometry of compounds.

Figure S3. Effect of licorice on the intestinal flora by 16s rDNA sequencing in mice (n = 10). (A) The Venn (ASV/OTUs); (B) The microbial α-diversity in seven groups; (C) The microbial β diversity calculated by PCoA; (D) The microbial β diversity calculated by NMDS; (E) LDA effect size (LEfSe) among seven groups; (F) The KEGG pathways. The dosages of Licorice (L), Licorice (M) and Licorice (H) were set at 0.4, 2 and 10 g (raw licorice)/(kg body weight) twice daily to mice.

Figure S4. The levels of uremic toxins in serum and in renal tissue. (A) Serum 3-(3,4-dihydroxyphenyl)-L-alanine (mg/mL); (B) Serum 1-methyl-inosine (ng/mL); (C) Serum N2, N2-Dimethylguanosine (ng/mL); (D) Serum N-acetylcytidine (ng/mL); (E) Serum Hippuric acid (ng/mL); (F) Renal phenylacetyl-L-glutamine (ng/mg); (G) Renal Indole-3-acetic acid (ng/mg); (H) Renal 3-(3,4-dihydroxyphenyl)-L-alanine level (ng/mg); (I) Renal 1-methyl-inosine (ng/mg); (J) Renal N2, N2-Dimethylguanosine (ng/mg); (K) Renal N-acetylcytidine (ng/mg); (L) Renal Hippuric acid (ng/mg). The dosages of Licorice (L), Licorice (M) and Licorice (H) were set at 0.4, 2 and 10 g (raw licorice)/(kg body weight) twice daily to mice. All data were presented as box-and-whisker plots (median, interquartile range, min-max). Non-parametric tests (Kruskal-Wallis H test with Dunn's post-hoc test) were used for statistical analysis of the data presented in Figures S4 (**C,D,F,G,J,K,L**). Data shown in Figures S4 (**A,B,E,H,I**) were analyzed by one-way ANOVA followed by Tamhane's T2 post-hoc test. $^{**}p < 0.01$, $^{*}p < 0.05$ vs. the control group; $^{\#\#}p < 0.01$, $^{\#}p < 0.05$ vs. the VAN group.
