# Supplementary material for: Protective Effects of Licorice (Glycyrrhiza uralensis) Against Vancomycin-Induced Nephrotoxicity In Vivo and In Vitro
Source: Pharmaceuticals (Basel). 2026 May 4;19(5):728. doi: 10.3390/ph19050728 (PMC13209765; doi:10.3390/ph19050728)
Supplement: Supplementary file 1 [file pharmaceuticals-19-00728-s001.zip › Table S1.pdf]

Table S1. Mass spectrometry detection conditions for four compounds.

Name	Precusor ion	Product ion	DP (V)	CE (V)	CXP (V)	Ion mode
liquiritin	417.0	135.0	-78.13	-36.9	-12.9	N
isoliquiritigenin	255.0	135.0	-45.97	-20.19	-8	N
quercetin	301.0	150.9	-71.01	-27.05	-11.02	N
liquiritin apioside	549.2	254.9	-117.69	-41.94	-16.03	N

Note: declustering potential (DP), collision energy (CE), collision cell exit potential (CXP), negative electrospray ionization (N).
