# Supplementary material for: Protective Effects of Licorice (Glycyrrhiza uralensis) Against Vancomycin-Induced Nephrotoxicity In Vivo and In Vitro
Source: Pharmaceuticals (Basel). 2026 May 4;19(5):728. doi: 10.3390/ph19050728 (PMC13209765; doi:10.3390/ph19050728)
Supplement: Supplementary file 1 [file pharmaceuticals-19-00728-s001.zip › Table S2.pdf]

Table S2. Mass spectrometry detection conditions for different uremic toxins.

Name	CAS	MW	MRM (m/z)	Frag (eV)	CE (eV)	Ion mode
phenylacetyl-L-glutamine	28047-15-6	264	263.6-145.4	100	4	N
indole-3-acetic acid	87-51-4	175	174.4-130.0	60	6	N
3-indoxyl sulfate	2642-37-7	251	212.4-80.0	90	14	N
hippuric acid	495-69-2	179	178.1-134.0	65	8	N
3-(3,4-dihydroxyphenyl)-L-alanine	59-92-7	197	198.0-151.9	80	8	P
1-methyl-inosine	2140-73-0	282	305.0-173.0	190	10	P
N2, N2-dimethylguanosine	2140-67-2	311	312.1-180	90	8	P
3-indolyl-β-D-glucopyranoside	487-60-5	295	318.0-256.1	120	18	P
N-acetylcytidine	3768-18-1	285	308.0-176.0	110	8	P
creatinine	60-27-5	113	114-44.2	70	16	P

Note: The instrument platform we used consists of an Agilent 1260 liquid chromatography system and a 6460 triple-quadrupole mass spectrometer, both purchased from Agilent Technologies (Santa Clara, CA, USA). Molecular weight (MW), multiple reaction monitoring (MRM), fragmentor voltage (Frag), collision energy (CE), negative electrospray ionization (N), positive electrospray ionization (P).
