# Supplementary figures and images for: Protective Effects of Licorice (Glycyrrhiza uralensis) Against Vancomycin-Induced Nephrotoxicity In Vivo and In Vitro
Source: Pharmaceuticals (Basel). 2026 May 4;19(5):728. doi: 10.3390/ph19050728 (PMC13209765; doi:10.3390/ph19050728)

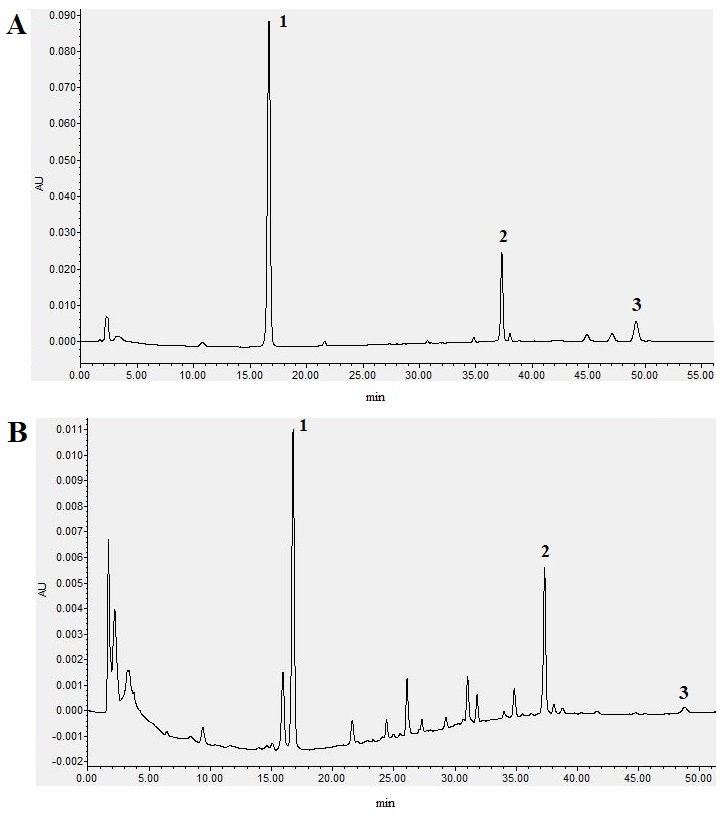

Supplement: Supplementary file 1 [file pharmaceuticals-19-00728-s001.zip › Figure S1.jpg]

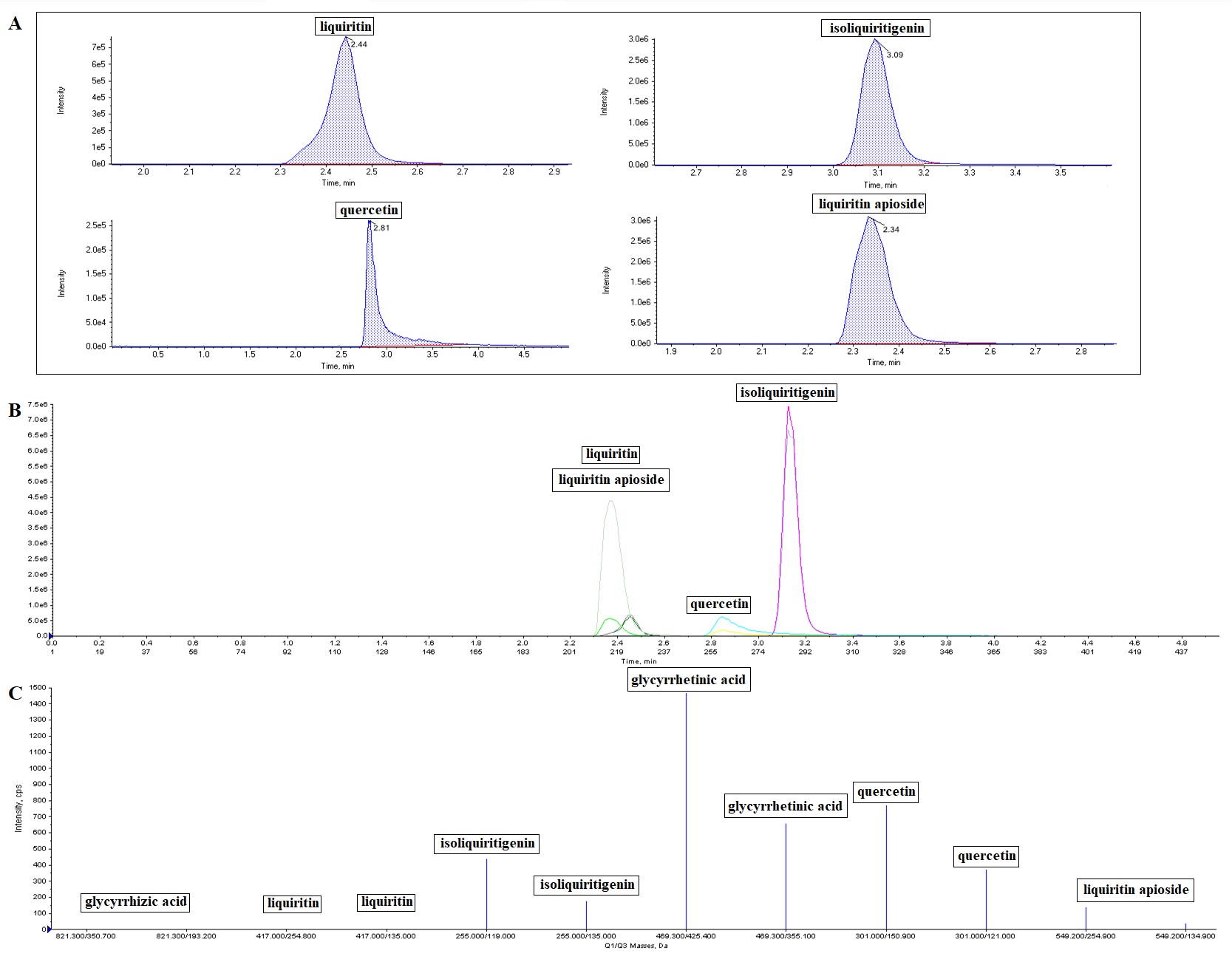

Supplement: Supplementary file 1 [file pharmaceuticals-19-00728-s001.zip › Figure S2.jpg]

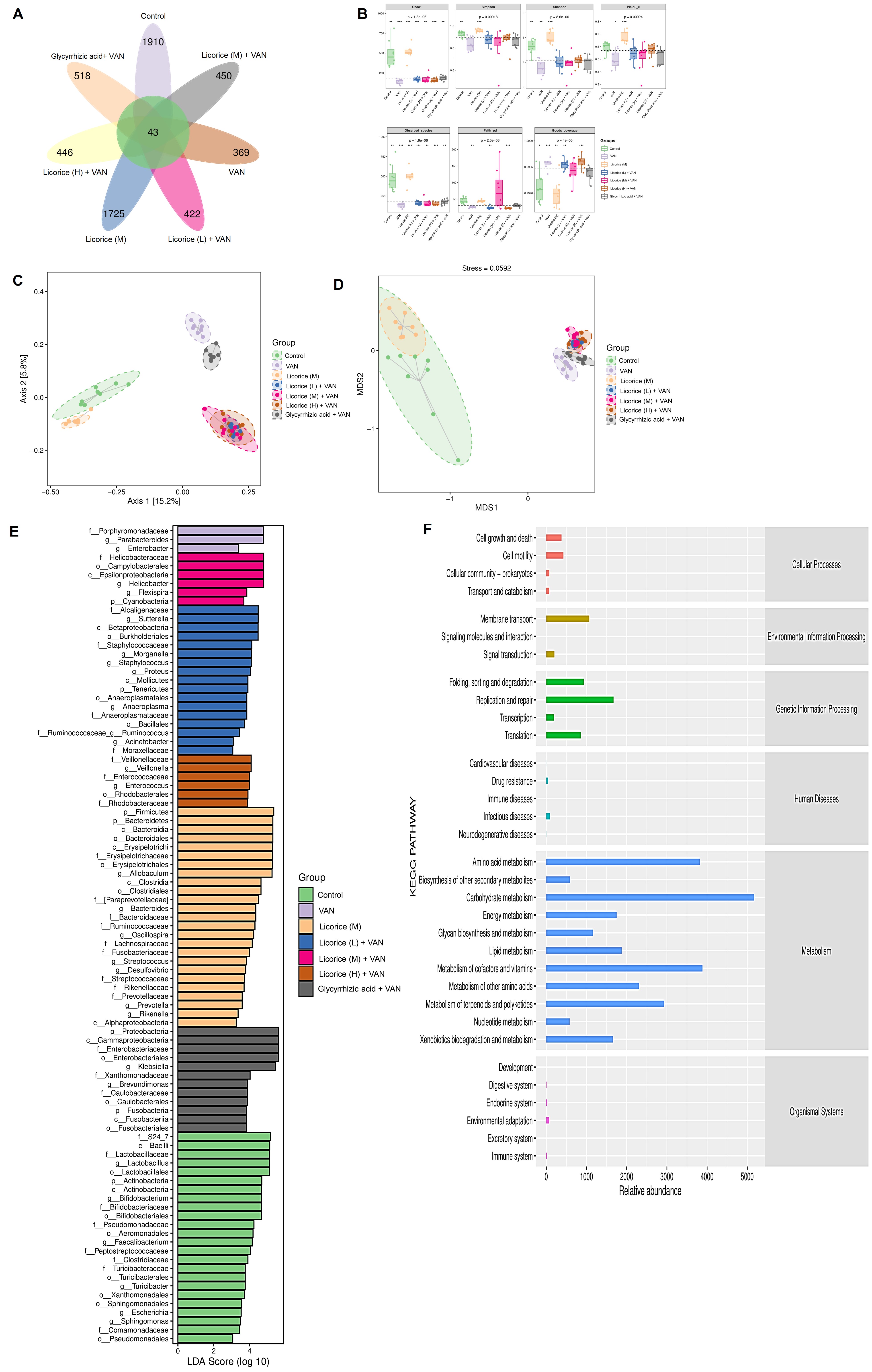

Supplement: Supplementary file 1 [file pharmaceuticals-19-00728-s001.zip › Figure S3.jpg]

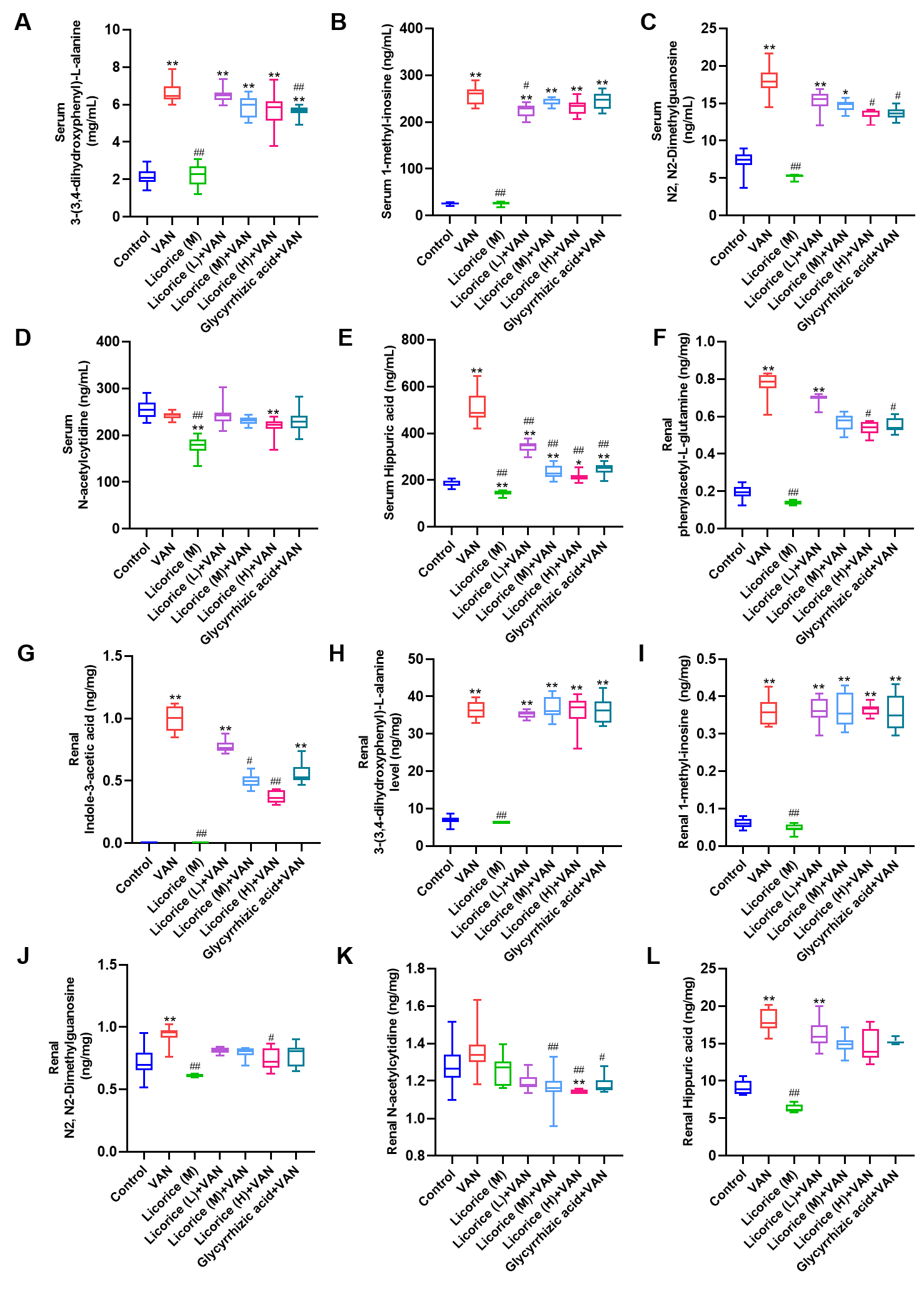

Supplement: Supplementary file 1 [file pharmaceuticals-19-00728-s001.zip › Figure S4.png]
